# The TMA team and TTP pathway improved outcomes in a cohort with Thrombotic thrombocytopenic purpura

**DOI:** 10.1371/journal.pone.0325417

**Published:** 2025-06-06

**Authors:** Samuel A. Merrill, Stephen Yu, Sylvia E. Webber, John Gotses, Emma L. Platt, Ruta Arays, Aaron D. Shmookler

**Affiliations:** 1 Department of Medicine, West Virginia University School of Medicine, Morgantown, West Virginia, United States of America; 2 Department of Oncology, West Virginia University School of Medicine, Morgantown, United States of America; 3 Section of Nephrology, Department of Medicine, West Virginia University School of Medicine, Morgantown, United States of America; 4 Department of Pharmacy, West Virginia University School of Medicine, Morgantown, West Virginia, United States of America; 5 Department of Internal Medicine, Division of Medical Oncology, University of Kentucky, Lexington, Kentucky, United States of America; 6 Department of Pathology and Laboratory Medicine, University of Kentucky, Lexington, Kentucky, United States of America; Tekirdag Namik Kemal University: Tekirdag Namik Kemal Universitesi, TÜRKIYE

## Abstract

**Background:**

Providing optimal care for patients with thrombotic thrombocytopenic purpura (TTP) is challenging because of multiple involved specialties, knowledge gaps, and a high rate of disease relapse. A thrombotic microangiopathy (TMA) Team and TTP Pathway could improve outcomes.

**Objectives:**

To assess if a structured TTP Pathway, supported by a TMA Team, improved TTP care by reducing TTP relapse and TTP-related death (TTP-RRD) at a rural Appalachian medical center.

**Methods:**

Prospective cohort quality improvement project using the DMAIC quality improvement framework (Define, Measure, Analyze, Improve, Control) to develop a TMA Team and TTP Pathway. Pathway care included standardized use of therapeutic plasma exchange (TPE), rituximab, caplacizumab, as well as improved coordination between medical services, and regular outpatient biochemical TTP surveillance. Outcomes were determined by retrospective chart review for patients with acute TTP treated with usual care (N = 16 episodes) and the TTP Pathway (N = 16 episodes).

**Results and conclusions:**

All patients had acquired TTP. TTP-RRD at 90 days was reduced from 69% with usual care to 6% with Pathway care (95% CI 0.35 to 0.90, P = 0.0004), a relative risk reduction of 91%; TTP relapse alone at 90 days was reduced from 62% to 0% (95% CI 0.36 to 0.88, P = 0.0002) with Pathway care. The number needed to treat to prevent TTP-RRD was 1.59 at 90 days. Over the project duration usual care demonstrated a hazard ratio for TTP-RRD of 12.58 compared to Pathway care. With the intervention, the duration of TPE was increased (median 6 vs 12 sessions, P < 0.05), as was use of rituximab (31.3% vs 93.8%, 95% CI −0.36 to −0.88, P = 0.003), and caplacizumab (6.3% vs 62.5%, 95% CI −0.027 to −0.81, P = 0.001). All Pathway patients underwent biochemical surveillance, and 31% had pre-emptive rituximab to reduce possibility of clinical relapse. A structured TTP Pathway significantly reduces morbidity and aligns care with modern clinical guidelines. The TMA Team is a valuable institutional resource to improve outcomes.

## Introduction

Thrombotic thrombocytopenic purpura (TTP) is a rare hematologic disorder characterized by excessive platelet activation due to ADAMTS13 deficiency, leading to microvascular thrombosis [1]. The majority of TTP patients have autoimmune TTP, from an acquired inhibitor of ADAMTS13, although inherited deficiency of ADAMTS13 can occur. Therapeutic plasma exchange (TPE) has been standard of care for acute TTP, along with adjunctive immune suppression with corticosteroids and rituximab for autoimmune TTP [2–5]. The addition of caplacizumab in acute TTP and active surveillance in remission to detect impending disease relapse can also improve outcomes [3,6–9]. Strategies are needed to improve acute TTP care, and to aggressively prevent TTP relapses in order to minimize the cumulative risk of death and disability. [10–13].

Although scoring systems such as PLASMIC and the French score can facilitate TTP diagnosis, scores have reduced utility in some populations, such as older individuals. [14,15]. The similarities between TTP and other thrombotic microangiopathies (can result in diagnostic uncertainty and delayed treatment, with resultant morbidity and mortality [16–18]. Many knowledge gaps and barriers exist to ideal TTP clinical care, and care is heterogeneous [19]. Knowledge gaps include the optimal duration of TPE, the use of a TPE taper, rituximab dose intensity, and frequency of surveillance testing to detect biochemical ADAMTS13 relapse before overt clinical relapse [2,9,20–22]. TTP care requires coordination across medical services (intensivists, proceduralists, hematology, blood bank/laboratory medicine, and often nephrology) and the inpatient-to-outpatient transition of care, which may lead to care delays and gaps in care. Furthermore, the TPE procedure may be conducted by hematology, transfusion medicine, or nephrology depending on local practices, potentially amplifying the clinical consequences of the knowledge gaps. Clinical pathways can improve care by structuring and standardizing multidisciplinary care for specific clinical problems [23,24].

To address these and other gaps in TTP care observed in our health system that contributed to poor outcomes, we formed a TMA Team as reported by Gordon et al., and subsequently implemented a TTP Pathway developed by the TMA Team [25]. We herein report our experience with the TTP Pathway at our center. The TTP Pathway is a multi-disciplinary effort to facilitate care and coordination, and establish evidence-based treatment and surveillance for TTP.

## Methods

This prospective cohort study was conceptualized with the DMAIC (define, measure, analyze, improve, control) methodology of Six Sigma [26–28]. Detailed methods are found in the supplement (S1 File). WVU Medicine is a hospital system providing care to patients in rural Appalachia within West Virginia, western Maryland, eastern Ohio, and southwestern Pennsylvania.

### Define

The primary outcome was defined as TTP relapse and TTP-related death (TTP-RRD) a priori. TTP relapse was defined as a second episode of active TTP with thrombocytopenia requiring therapy occurring in a patient with a prior episode of acute TTP in the study period (equivalent to “clinical exacerbation” and “clinical relapse” as defined by consensus definition subsequently) [16,29,30]. Other definitions are noted in S1 File.

Inclusion criteria: age ≥ 18 years, with a diagnosis of new TTP or relapsed TTP. Exclusion criteria: age < 18, other TMA determined to not be TTP at the discretion of the treating physicians and during chart review, or missing data preventing TTP diagnosis. A priori TTP-RRD was the primary outcome. Secondary outcomes defined a priori were TTP-related death, TTP relapse, use of rituximab in acute TTP, and use of pre-emptive rituximab during clinical remission intended to prevent TTP clinical relapse.

### Measure

A process map was done for TTP hospitalization and outpatient care. Areas of need were identified by TMA Team members and retrospective chart review of cases between 2017 and 2019.

### Analyze

Causes of TTP-RRD in the pre-Pathway time period were analyzed with an events and causal factor analysis using an Ishikawa diagram [27]. The following areas of need were identified: having a TTP expert resource (TMA Team [25]), educational materials for care providers, adding caplacizumab to hospital formulary, arranging follow up with hematology clinic post-discharge, developing a standardized TPE and rituximab use protocol, and conducting ADAMTS13 active surveillance to detect biochemical relapses before overt TTP has developed [27].

### Improve

To systematically address needs with our limited resources the following changes were adopted: formation of a volunteer TMA Team, generation and distribution of resident and consult service educational materials in the form of pocket cards (S2 File and S3 File), establishing the TPE and immunosuppression protocol with planned duration of TPE and expected rituximab use, arranging outpatient follow up with hematology before discharge from hospital, planned outpatient surveillance of ADAMTS13 activity no less than every 3 months, obtaining caplacizumab on formulary and developing recommendations for its use.

The TPE protocol was modified from Rock et al. (S1 File) [4]. TPE was expected to use 12–14 sessions, however duration was to be determined by the treating hematologist at outpatient follow up (SAM, SY, and RA). If caplacizumab was used, TPE would be truncated, as in the trial protocol [7]. Details of apheresis line placement, biochemical surveillance, and rituximab use in acute TTP are noted in the supplement (S1 File). The TTP Pathway became active in November 2019 (S1 Fig). Although we found education ineffective at improving quality metrics in a rare disease previously, direct education and pocket cards were used to raise awareness of the Pathway at the academic center to encourage TMA Team involvement [31].

### Control

TTP-RRD was assessed by retrospective analysis yearly or when an event occurred. Failures were analyzed with an events and causal factor analysis to determine if intervention process changes were needed consistent with the reactive poka-yoke principle [27]. During the project, ISTH guidelines on TTP were published and encouraged caplacizumab use, thus the TMA Team further encouraged caplacizumab use [2].

### Data acquisition

Retrospective chart review was conducted to assess outcomes. The inclusion criteria and exclusion criteria are listed above. The time period for analysis was Jan 1, 2016—Dec 31, 2023. The TTP Pathway became operational on 11/11/2019. Patients with ADAMTS13 activity test results <30% were identified from the electronic health records and assessed by chart review. Two hematology physicians (SY and SAM) reviewed cases and determined if patients had TTP by consensus (S1 File). Data were accessed for research purposes on April 10, 2024, and again to address reviewer requests April 15–18, 2025. Only authors collecting data (SAM and SY) had access to information that could identify individual participants during data collection before de-identification. Patients were included from admission date for the first acute TTP episode occurring in the study window. Clinical information was then abstracted for TTP episodes. Area Deprivation Index (ADI) for 2022 was obtained for each patient [32,33]. Patient episodes treated on the TTP Pathway were identified from the prospectively maintained administrative database. This study was approved by the Institutional Review Board of West Virginia University (Protocol #: 2012188025 and 2503120932). Informed consent was waived by the Institutional Review Board of West Virginia University. This study is presented following STROBE guidelines [34].

### Statistical analysis

A priori the primary and secondary outcomes were to be assessed by log-rank testing, and by proportion analysis at 90 days. Statistical significance was assessed using confidence intervals and effect sizes rather than p-values alone, following contemporary recommendations on statistical reporting. Relative risks (RR) and hazard ratios (HR) with 95% confidence intervals were calculated where applicable. Given the rarity of TTP, a formal power analysis was not feasible, but all available cases meeting inclusion criteria were included. Kaplan-Meier survival analysis, log rank testing, and Wilcoxon rank sum test were used in comparisons where indicated. Statistical significance was set at P < 0.05 with two-tailed testing, and 95% confidence intervals were calculated for comparisons. Computations utilized STATA 18, (StataCorp. 2023. Stata Statistical Software: Release 18. College Station, TX).

## Results

Fifty-eight patients were identified by ADAMTS13 testing. Thirty-two patients were excluded for not having TTP. Non-TTP etiologies were: disseminated intravascular coagulation in the setting of malignancy, infection, or vasculitis (N = 11), infection or sepsis (N = 12), missing data (N = 3), lupus or vasculitis (N = 2), drug induced thrombocytopenia (N = 1), cirrhosis (N = 1), other TMA (N = 1), and history of TTP without acute TTP in study window (N = 1). The remaining 26 patients with at least 1 acute TTP episode in the study period were included for analysis: 13 patients received usual care, 16 patients received TTP Pathway care; and 3 patients received both usual care and at time of relapse were subsequently treated on the TTP Pathway. Patient characteristics are listed in Table 1. TTP patients were predominantly women, white race, and the majority had a history of previous TTP before the study period. Socioeconomic and geographic barriers to care were common. There were more new TTP episodes in the Pathway cohort, yet the difference was not significant (95% CI −0.28 to 0.36, P = 0.781). The majority of patients (72.5%) were in the bottom third of affluence for the nation by area deprivation index (ADI). There were two cases of COVID-19 associated TTP. All patients had autoimmune TTP, and no cases of congenital TTP were identified.

**Table 1:** Demographic data.

	All patients		Usual Care		TTP Pathway	
Patient Demographic	No. (%)	Median (range)	No. (%)	Median (range)	No. (%)	Median (range)
Age, yrs	26/26 (100)	40 (23-81)	13/26 (50)	38 (24-74)	16/26 (61.5)	45 (23-81)
Sex, male	7/26 (36.8)	–	5/13 (38.4)	–	2/16 (12.5)	–
Follow up, days	26/26	677 (7-2226)	13/13	166 (7-2226)	16/16	699 (7-1511)
Race						
White	23/26 (88.5)	–	11/13 (84.6)	–	15/16 (93.8)	–
Black	2/26 (7.7)	–	2/13 (15.4)	–	0/16 (0)	–
Asian	1/26 (3.8)	–	0/13 (0)	–	1/16 (6.3)	–
Distance from site (mi)	26/26 (100)	82.5 (15-1024)	13/13 (100)	86.6 (32-1024)	16/16 (100)	82.5 (15-198)
>50 miles	22/26 (85)	–	10/13 (77)	–	12/16 (75)	–
Prior TTP episode	14/26 (53.8)	–	8/13 (61.5)	–	9/16 (56.3)	–
Medicare/Medicaid	12/26 (46.2)	–	5/13 (38.5)	–	8/16 (50.0)	–
ADI	26/26 (100)	72.5 (2-95)	13/13 (100)	70 (8-88)	16/16 (100)	77 (2-95)
ADI most deprived (67–100)	19/26 (73.1)	–	8/13 (61.5)	–	11/16 (68.8)	–
**Acute TTP Therapy**	**No.**	**%**	**No.**	**%**	**No.**	**%**
Corticosteroid	32/32	100.0%	16/16	100.0%	16/16	100.0%
Rituximab	20/32	62.5%	5/16	31.3%	15/16	93.8%
Caplacizumab	11/32	34.4%	1/16	6.3%	10/16	62.5%
**Episode characteristic**	**No. (%)**	**Median (range)**	**No. (%)**	**Median (range)**	**No. (%)**	**Median (range)**
Hgb, g/dL	32/32 (100)	10.4 (3.6-13.5)	16/16 (100)	11.1 (5.4-13.5)	16/16 (100)	9.9 (3.6-13.4)
PLT, x 10^9/L	32/32 (100)	15.5 (0-99)	16/16 (100)	15.5 (4-99)	16/16 (100)	15.5 (0-32)
LDH, U/L	31/32 (97)	1000 (303-5124)	16/16 (100)	1041 (303-2756)	15/16 (94)	1000 (364-5124)
Cr, mg/dL	32/32 (100)	1.1 (0.6-3.8)	16/16 (100)	1.2 (0.9-3.8)	16/16 (100)	1.1 (0.6-2.9)
ADAMTS13 activity, % *	31/32 (97)	<5% (<5-36)	16/16 (100)	<5% (<5-36)	15/16 (94)	<5% (<5-36)
TPE use and duration, days	31/32 (97)	9 (3-29)	15/16 (94)	6 (3-29)	16/16 (100)	11.5 (4-22)
TPE duration, no caplacizumab	20/31 (65)	6.5 (3-29)	14/15 (93)	5.5 (3-29)	6/16 (38)	15 (4-22)
Admission to TPE, days	31/31 (100)	1 (0-7)	15/15 (100)	1 (0-7)	16/16 (100)	1 (0-3)
TPE to PLT 150, days**	28/31 (90)	4.5 (2-40)	13/15 (87)	4 (2-40)	15/16 (94)	5 (2-11)
First episode of TTP	12/32 (38)	–	5/16 (31)	–	7/16 (44)	–
Against Medical Advice	2/32 (6.3)	–	2/16 (13)	–	0/16 (0)	–
Presented to community site	16/32 (50)	–	9/16 (56)	–	10/16 (63)	–
Presented to academic site	16/32 (50)	–	7/16 (44)	–	6/16 (38)	–

TTP: thrombotic thrombocytopenic purpura; TPE: therapeutic plasma exchange; Hgb: hemoglobin; PLT: platelet; LDH: lactate dehydrogenase; Cr: creatinine; ADI: Area Deprivation Index. * Includes TTP relapse with higher ADAMTS13 activity than presenting episode. ** Patients who died did not attain PLT 150.

Usual care and TTP Pathway care were notably different (Table 1). The use of rituximab in acute TTP was increased with the TTP Pathway (95% CI −0.36 to −0.88, P = 0.003), as was use of caplacizumab (95% CI −0.27 to −0.81, P = 0.001). However, caplacizumab was not available for 3 of the 4 years in the study before the TTP Pathway began. The number of TPE sessions was significantly higher on the TTP pathway (median 6 vs 12, P = 0.0011 by two sample Wilcoxon rank sum test), despite the frequent use of caplacizumab that enabled shorter TPE duration. Limiting analysis to non-caplacizumab treated episodes, TPE duration was longer with TTP Pathway care (median 5.5 vs 15, P = 0.027 by two sample Wilcoxon rank sum test). All patients on the TTP Pathway received rituximab during the acute TTP episode, except 1 patient that died with acute TTP while receiving daily TPE (Table 1). There was no difference between usual care and TTP Pathway care in: corticosteroid use, presentation at the academic center versus community hospitals (95% CI −0.40 to 0.28, P = 0.721), having had TPE (95% CI −0.18 to 0.056, P = 0.31), or leaving hospital against medical advice (95% CI −0.037 to 0.29, P = 0.14).

**Fig 1:**
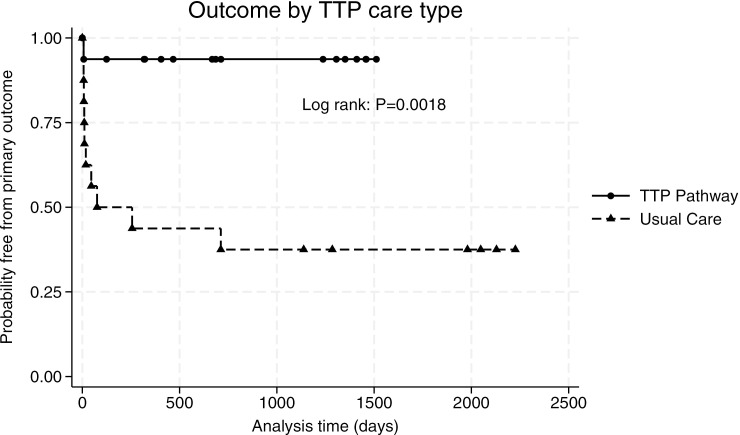
Primary outcome of TTP-RRD. Kaplan-Meier analysis of all patients with acute TTP treated with usual care or TTP Pathway care during the study period, with long-rank testing indicated.

The primary outcome of TTP-RRD at 90 days occurred in 69% of patients with usual care versus 6% of patients on the TTP pathway (95% CI 0.35 to 0.90, P = 0.0004) (Table 2). The relative risk of TTP-RRD at 90 days was higher with usual care (RR 9.0, 95% CI 1.28 to 63.02, P = 0.006), and the NNT for benefit on the TTP Pathway was 1.59. The TTP-RRD relative risk reduction of 91.3% met the pre-specified outcome for pathway success (50%); TTP-RRD absolute risk reduction was 63%. The TTP Pathway attained the secondary objective of increased use of rituximab for acute TTP (RR 3, 95% CI 1.43 to 6.27, P = 0.0006). Four patients died of acute TTP, three from the usual care cohort (S1 Table). TTP-related death was reduced (RR 0.27, 95% CI 0.032 to 2.3), but not significantly different between usual care and TTP pathway care. TTP relapse at 90 days was reduced with TTP pathway care (62% vs 0%, 95% CI 0.36 to 0.88, P = 0.0002; RR 0, 95% CI not estimable due to zero events, P = 0.002), and the 90 day relapse NNT was 2.8. During biochemical surveillance on the TTP Pathway, 31% of patients were preemptively treated with rituximab due to declining ADAMTS13 activity with the intent to prevent TTP relapse, no patients were treated with preemptive rituximab before the 90 day analysis period. No patients in the usual care group had ADAMTS13 surveillance or preemptive rituximab therapy (95% CI −0.083 to −0.54, P = 0.015). TTP Pathway care also significantly reduced the primary outcome of TTP-RRD over the entire study period (Fig 1, log-rank P = 0.0018). The HR for TTP-RRD with usual care during the project was 12.58 (95% CI 1.61 to 98.67, P = 0.016) compared to TTP Pathway care. Median distance from the academic center to patient was 82 miles, and 85% of patients were more than 50 miles from the academic center. Distance >50 miles from the center was not associated with TTP-RRD at 90 days in the usual care group (95% CI −0.27 to 0.75, P = 0.38), however numbers were small. Newly diagnosed TTP can have increased mortality as a confounding factor, and more new TTP cases were seen on the TTP Pathway [35,36]. In subgroup analysis, the proportion of newly diagnosed TTP patients with TTP-RRD was higher with usual care (0.60 vs 0.14, 95% CI 0.04 to −0.96, P = 0.095) but the difference was not statistically significant given small numbers for comparison (RR 4.2, CI 0.60 to 29.5, P = 0.22; log-rank P = 0.25) (S2 Fig).

**Table 2:** TTP Pathway Outcomes.

Outcome Measure	Usual Care	TTP Pathway	95% CI	P-value
Proportion with TTP-RRD at 90 days	0.69	0.06	0.35 to 0.90	0.0004
TTP-death	0.23	0.06	−0.09 to 0.43	0.18
TTP-relapse	0.62	0.00	0.36 to 0.88	0.0002
Proportion treated with rituximab (acute TTP)	0.31	0.94	−0.89 to −0.38	0.0002
Proportion with preemptive rituximab	0.00	0.31	−0.083 to −0.54	0.015
Post-acute TTP outpatient follow up site				
Academic center (+/- local oncology)	0.58	0.93	−0.66 to −0.42	0.031
Local oncologist only	0.17	0.07	−0.15 to 0.35	0.42
None	0.25	0.00	0.005 to 0.49	0.04

Outpatient TTP management site of care differed with the TTP Pathway. Academic center follow-up with classical hematology was increased (58% vs 93%, 95% CI −0.66 to −0.42, P = 0.031; RR 1.6, 95% CI 0.97 to 2.63), and fewer surviving patients had no outpatient follow up after the acute TTP episode (25% vs 0%, 95% CI 0.005 to 0.49, P = 0.04; RR 0, 95% CI not estimable due to zero events). Duration of caplacizumab therapy with academic center follow-up was 1 month after hospital discharge for all TTP Pathway patients based on ADAMTS13 recovery, except for the patient who declined follow-up: that patient was treated locally for 3 months and outpatient ADAMTS13 data were not available. On the TTP pathway, 50% of patients treated with caplacizumab experienced some adverse event attributed to caplacizumab including: new onset heavy menses (N = 2); severe needle phobia with injection anxiety (N = 2, required caregiver to administer injections), mild epistaxis (N = 1); one patient had “profuse” bleeding at the site of femoral venous access 4 hours after temporary apheresis catheter removal with a platelet count of 275, this required pressure and a dressing change. No adverse event required caplacizumab discontinuation.

Caplacizumab availability and use at regional sites was explored for acute therapy of clinically suspected TTP before patient transfer to the academic center for TPE. However, due to low utilization and cost concerns at regional sites this was ultimately not feasible. During the project the use of caplacizumab became more common over time, as a result of ISTH guideline recommendations [2]. As part of the *Control* process, we did observe that inpatient physicians were often using caplacizumab with the standard anticipated TPE duration of 12–14 sessions, which was not intended. Pathway clarification on TPE duration was attained with the TMA Team pharmacist who rounded with the consult service. Other difficulties were limited clinical follow-up appointment availability for care continuity due to limited providers in classical hematology, onerous prior authorizations for caplacizumab, initial difficulty obtaining caplacizumab (adding to formulary, pharmacy availability), delay in obtaining a hematology pharmacist for the team, and TMA Team clinical effort was not directly supported. At our institution medications given at home (caplacizumab) need authorization by the prescribing physician, which was an additional burden on the consult team until the addition of the pharmacist.

## Discussion

Acute TTP is a rare and life-threatening medical emergency with substantial morbidity for survivors [12,13,37]. TTP care can be difficult to coordinate because of the number of medical services involved and knowledge gaps that affect clinical decisions. Using a multidisciplinary approach to TTP care with a TMA Team, we developed a TTP Pathway by identifying areas for improvement in usual care. The TTP Pathway was associated with significant and clinically meaningful improvements in patient care, such as a 91% relative reduction in TTP-RRD at 90 days, a NNT of 1.59 to prevent one episode of TTP-RRD at 90 days, and routine use of rituximab in acute TTP that was later recommended in guidelines [2]. The negative clinical outcome of TTP-RRD was markedly reduced with the TTP Pathway (RR 9.0 with usual care). Over the duration of the project a marked reduction of TTP-RRD was observed (HR 12.58 with usual care). Although TTP mortality was meaningfully reduced with the Pathway (23% vs 6%, RR 0.27), despite more initial TTP episodes in this cohort where mortality can be higher, this improvement was not statistically different from usual care with our small patient numbers [35,36].

Novel TTP treatments (caplacizumab) can improve TTP outcomes, but more effective use of existing treatments (TPE, immune suppression) and care coordination (TMA Team, ADAMTS13 surveillance) can also be beneficial [2,3,7,25,38,39]. Improving care delivery could be valuable in both high and low resource settings, and care pathways can help ensure use of guideline-based care. [17,19] Although TMA Teams are found at many academic centers, there is an absence of data showing the effectiveness of a TMA Team, except for one report on non-TTP TMA [40]. Our TTP Pathway streamlined care and demonstrated clear clinical benefit of the TMA Team, but barriers to care were noted during the project.

Our catchment area is rural and underserved. In this study 72.5% of TTP patients are in the bottom third of affluence in the nation by ADI. Sub-specialist classical hematology and TPE are available at only one site in the region. We observed that use of caplacizumab greatly facilitated TTP transitions of care and outpatient management, where many patients had difficulty traveling for TPE. The ISTH TTP guideline recommends routine use of caplacizumab in acute TTP, however this was controversial due to concerns about hemorrhage and cost [41,42]. Caplacizumab is expensive and was not cost-effective on analysis [43]. However, routine and early use of caplacizumab reduced TPE refractory TTP, can shorten hospital stays, can shorten TPE, and can be cost saving compared to delayed caplacizumab use or non-use [7,43,44]. We believe that routine caplacizumab use can specifically benefit rural and underserved patients who face disproportionate barriers to outpatient TPE, while also reducing relapses when used with upfront immune suppression. [44] The possibility of TPE-free TTP treatment would represent a considerable therapeutic advance, especially for rural and underserved patients where TPE is a disproportionate barrier to care. Although there are initial reports on TPE-free treatment, further studies are needed to demonstrate clinical effectiveness and cost effectiveness before this would become a standard treatment [45].

The ideal duration of TPE is not known. The benefit of a TPE taper is uncertain. Despite these knowledge gaps taper use was common in the USA and Canada in the pre-caplacizumab era, and many TPE cessation strategies were reported [46,47]. TTP guidelines either do not comment on TPE duration or taper, or recommend TPE until 2–3 days after platelet normalization, yet studies specifically analyzing TPE cessation strategies are lacking [2,5,48]. ADAMTS13 recovery after immune suppression and TPE may take 1–2 weeks or longer [3,44]. Longer duration TPE can function as a bridge in this window via antibody depletion. Indeed, others observed a significant delay in ADAMTS13 recovery with caplacizumab, attributed to less TPE and rituximab use [49,50]. The high TTP recurrence rate of 38% in the HERCULES trial within 30 days for non-caplacizumab treated patients supports this idea. The short reported TPE duration (median 7 days), infrequent rituximab use (48%) and high recurrence rates were similar to our observations for usual care, which prompted this improvement project [7]. Our usual care TPE duration of 6.5 days was shorter than other studies (median 10 in Coppo et al.; median 15 and 17 in Gomez-Segui et al.; mean 13 in Radhwi et al; median 11 in Page et al.), but similar to some studies (median 7 Scully et al.; median 5 in Van de Louw, et al.) [7,37,51–53]. Given our prior experience (S1 File) and studies suggesting improved responses with more TPE or taper use, the TTP Pathway focused on either extending TPE in conjunction with rituximab immunosuppression or using caplacizumab in conjunction with rituximab immunosuppression [21,22,54]. Future studies on TPE length or tapers may be moot or not feasible with increasing use of upfront caplacizumab and rituximab as reported here and as in Coppo et al., except in low resource settings where cost concerns may be prohibitive [44].

This report shows that TTP outcomes can be meaningfully improved with a TMA Team and TTP Pathway. We show that the TMA Team provides demonstrable benefit in our health system. Similar approaches can improve outcomes at other centers for TTP. Our study has notable limitations. First, our TMA Team is not a formal or supported entity, and all inpatient therapy was at the discretion of the treating physicians (typically solid tumor oncologists). As such, care heterogeneity was expected, as observed with TPE tapers in patients treated with caplacizumab, where no taper was intended. Because of this, our study was neither designed nor intended to be a comprehensive trial on the total use and function of the TMA Team. Second, confounding did occur because of the multiple interventions used on the Pathway. This was by design with the DMAIC method, and is distinct from a randomized trial. The events and causal factor analysis determined that multiple interventions were required simultaneously to maximally reduce the critical to quality characteristic of TTP-RRD (S1 File), therefore we cannot determine the relative effectiveness of each intervention. Third, this Pathway was specific for TTP, and presence of another TMA would require a distinct care path. However, because of the nature of the intervention, the rarity of TTP cases, and small patient numbers utilizing the TTP Pathway, the TTP Pathway was much easier to maintain than a similar intervention for hemophilia patients, where direct clinical involvement was substantial and ultimately not sustainable [55]. Finally, TTP is a rare disease and as expected in a single-institution setting for a rare disease our patient numbers are small, although this is the first report of a TMA Team changing TTP outcomes to our knowledge.

## Conclusions

The use of a TTP Pathway incorporating evidence-based care, coordination between specialties, and routine surveillance improved TTP care and reduced relapses. Importantly, TTP Pathway care addressed distinct disease-specific needs during acute management in hospital and in the ambulatory clinic. This Pathway was easy to maintain after development and required only minimal clinical effort for continuation. Using a quality improvement approach to address barriers to care can improve outcomes in TTP, and for other hematologic disorders at other centers. The TMA Team has an important role in ensuring that TMA clinical care continues to improve as knowledge and therapies evolve.

## Supporting information

S1 Fig Graph showing TTP episodes by month of the study. Patients treated on TTP Pathway and usual care are noted. TTP Pathway became operational November 2019.
(DOCX)

S2 Fig Kaplan-Meier analysis of new TTP presentations. Patients treated on TTP Pathway and usual care with newly diagnosed acute TTP, with long-rank testing indicated.
(PDF)

S1 Table Individual patient level data. (PDF)

S1 File Supplemental Methods. (DOCX)

S2 File Fellow TMA Card. Educational guide distributed to fellows on the Hematology/Oncology consult service.
(PDF)

S3 File Resident TMA Card. Educational guide available to all staff via institutional intranet website, intended for internal medicine resident audience.
(PDF)
