# Supplementary material for: The TMA team and TTP pathway improved outcomes in a cohort with Thrombotic thrombocytopenic purpura
Source: PLoS One. 2025 Jun 6;20(6):e0325417. doi: 10.1371/journal.pone.0325417 (PMC12143514; doi:10.1371/journal.pone.0325417)

**S1 Fig. Graph showing TTP episodes by month of the study**. Patients treated on TTP Pathway and usual care are noted. TTP Pathway became operational November 2019.


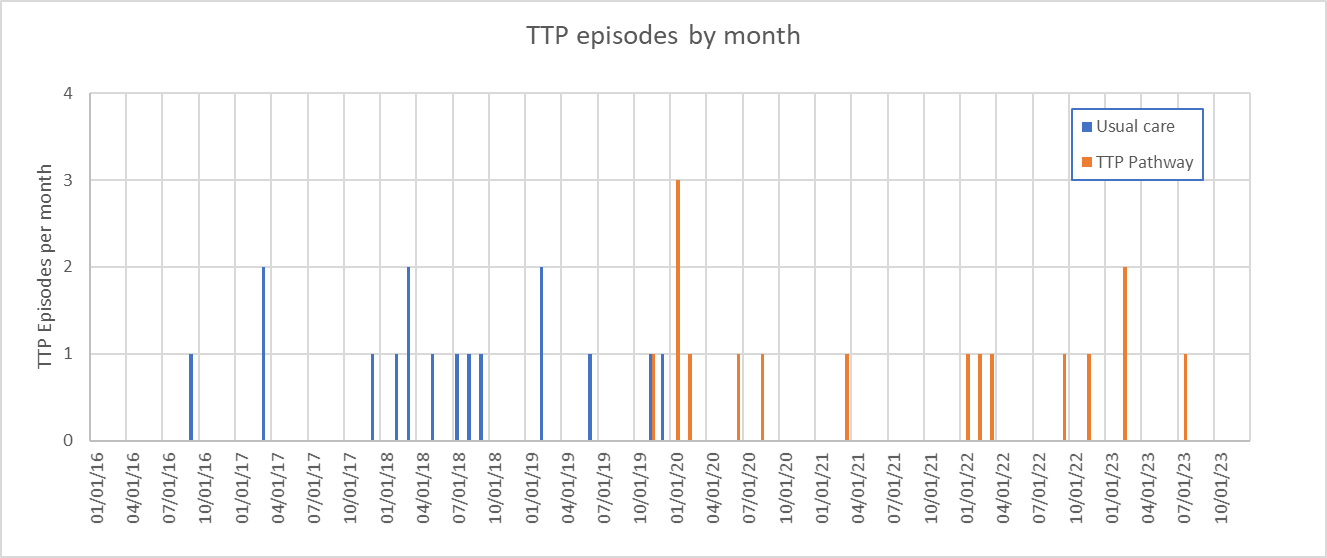

Supplement: S1 Fig — Patients treated on TTP Pathway and usual care are noted. TTP Pathway became operational November 2019. (DOCX) [file pone.0325417.s001.docx]
