# Supplementary figures and images for: The TMA team and TTP pathway improved outcomes in a cohort with Thrombotic thrombocytopenic purpura
Source: PLoS One. 2025 Jun 6;20(6):e0325417. doi: 10.1371/journal.pone.0325417 (PMC12143514; doi:10.1371/journal.pone.0325417)

## New TTP Diagnosis Only

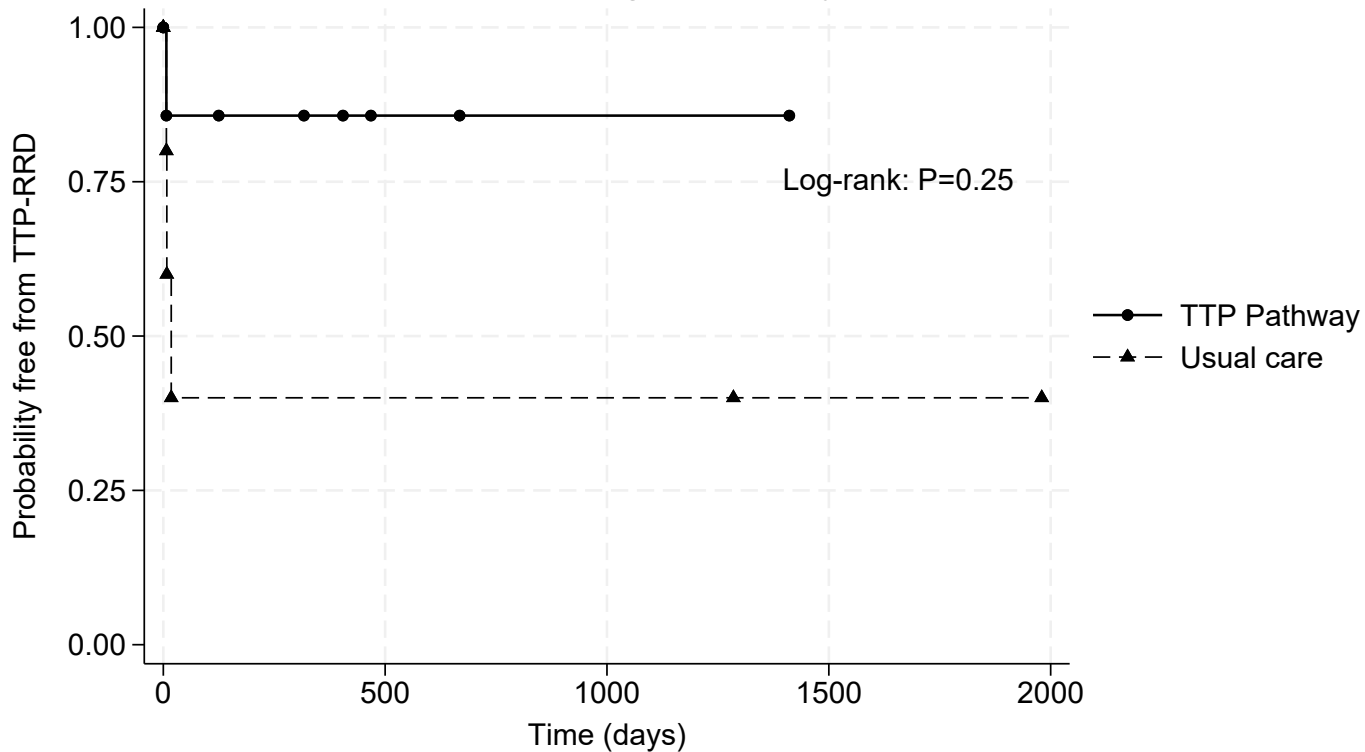

Supplement: S2 Fig — Patients treated on TTP Pathway and usual care with newly diagnosed acute TTP, with long-rank testing indicated. (PDF) [file pone.0325417.s002.pdf]
