# Supplementary material for: The TMA team and TTP pathway improved outcomes in a cohort with Thrombotic thrombocytopenic purpura
Source: PLoS One. 2025 Jun 6;20(6):e0325417. doi: 10.1371/journal.pone.0325417 (PMC12143514; doi:10.1371/journal.pone.0325417)
Supplement: S1 Table — (PDF) [file pone.0325417.s003.pdf]

S1 Table. Individual patient level data.

Cohort	Patient ID	Age	Sex	New vs Relapse	Acute TTP Medical Therapy	TPE (Y/N)	TPE sessions	Adm Hgb	Adm Plt	Adm LDH	Adm Cr	ADAMTS13 (%)	TTP-RRD (any)	Time to event or last follow up (days)	TTP-RRD at day 90	TTP death	Cause of death
Intervention	11.3	39	F	Relapsed	corticosteroid, rituximab	Y	10	13.4	0	-	1.18	-	no	1511	no	no	N/A
Intervention	14.1	32	F	Relapsed	corticosteroid, rituximab	Y	14	13	31	364	0.63	1	no	1459	no	no	N/A
Intervention	13.2	35	F	Relapsed	corticosteroid, rituximab, caplacizumab	Y	14	8.8	16	1611	1	36	no	1458	no	no	N/A
Intervention	15.1	37	F	Relapsed	corticosteroid, rituximab, caplacizumab	Y	9	10	11	1000	2.86	1	no	1351	no	no	N/A
Intervention	16.1	65	F	New	corticosteroid, rituximab, caplacizumab	Y	17	9.7	21	861	0.77	1	no	1411	no	no	N/A
Intervention	17.1	61	F	Relapsed	corticosteroid, rituximab	Y	17	9.1	16	876	0.81	1	no	1306	no	no	N/A
Intervention	8.2	31	F	Relapsed	corticosteroid, rituximab	Y	22	12.3	21	676	0.95	6	no	1238	no	no	N/A
Intervention	18.1	58	M	New	corticosteroid	Y	4	5.9	3	1316	1.04	8	yes	7	yes	yes	Refractory TTP with GI hemorrhage and cardiac arrest
Intervention	19.1	81	F	Relapsed	Pednisone, rituximab	Y	16	12.1	10	852	1.29	1	no	712	no	no	N/A
Intervention	20.1	23	F	Relapsed	corticosteroid, rituximab, caplacizumab	Y	22	12.1	8	1584	1.32	1	no	685	no	no	N/A
Intervention	21.1	79	F	New	corticosteroid, rituximab, caplacizumab	Y	9	10.2	27	752	1.98	1	no	668	no	no	N/A
Intervention	22.1	54	M	New	corticosteroid, rituximab, caplacizumab	Y	12	8.2	19	2346	1.16	1	no	468	no	no	N/A
Intervention	23.1	41	F	New	corticosteroid, rituximab, caplacizumab	Y	11	5.1	15	5124	0.6	11	no	405	no	no	N/A
Intervention	24.1	49	F	Relapsed	corticosteroid, rituximab, caplacizumab	Y	7	11.7	32	921	0.92	1	no	321	no	no	N/A
Intervention	25.1	62	F	New	corticosteroid, rituximab, caplacizumab	Y	7	8.2	8	1421	1.17	1	no	317	no	no	N/A
Intervention	26.1	34	F	New	corticosteroid, rituximab, caplacizumab	Y	9	3.6	8	1415	1.46	10	no	125	no	no	N/A
Usual care	1.1	50	F	Relapsed	corticosteroid	Y	7	11.2	11	1998	3.84	1	no	2129	no	no	N/A
Usual care	2.1	37	M	New	corticosteroid	Y	4	8.7	11	1217	1.30	1	yes	18	yes	no	N/A
Usual care	2.2	37	M	Relapsed	corticosteroid, rituximab	Y	3	12.2	99	303	1.07	36	no	2226	no	no	N/A
Usual care	3.1	33	F	New	corticosteroid	Y	6	10	7	2,756	1.30	1	no	1285	no	no	N/A
Usual care	4.1	24	M	New	corticosteroid	Y	3	13.2	18	1144	1.20	1	yes	7	yes	no	N/A
Usual care	4.2	24	M	Relapsed	corticosteroid	Y	5	5.4	14	1121	1.02	1	yes	11	yes	yes	TTP relapse with cardiac arrest
Usual care	5.1	74	M	Relapsed	corticosteroid	N	0	11.2	44	606	1.42	11	yes	76	yes	no	N/A

Usual care	6.1	48	F	Relapsed	corticosteroid	Y	10	10.3	21	575	1.42	29	no	2048	no	no	N/A
Usual care	7.1	73	M	New	corticosteroid	Y	7	8.4	9	509	0.96	5	no	1980	no	no	N/A
Usual care	8.1	29	F	Relapsed	corticosteroid, rituximab	Y	29	13.5	34	795	0.89	6	yes	712	no	no	N/A
Usual care	9.1	59	F	New	corticosteroid, rituximab	Y	9	12.8	16	1,761	1.50	1	yes	8	yes	yes	Refractory TTP with cardiac arrest
Usual care	10.1	58	M	Relapsed	corticosteroid	Y	3	10.9	15	1977	2.30	1	yes	46	yes	yes	TTP relapse with stroke and cardiac arrest
Usual care	11.1	30	F	Relapsed	corticosteroid	Y	5	12.3	25	689	1.10	7	yes	256	yes	no	N/A
Usual care	12.1	60	F	Relapsed	corticosteroid, rituximab, caplacizumab, vincristine	Y	13	10.5	6	1,460	1.08	1	no	1138	no	no	N/A
Usual care	11.2	39	F	Relapsed	corticosteroid	Y	6	12.1	46	461	1.13	14	yes	10	yes	no	N/A
Usual care	13.1	35	F	Relapsed	corticosteroid, rituximab	Y	4	8.5	4	962	0.92	7	yes	7	yes	no	N/A

Adm: admission laboratory value, units per Table 1. N/A: not applicable. Y/N: Yes/No.
