# Supplementary material for: The TMA team and TTP pathway improved outcomes in a cohort with Thrombotic thrombocytopenic purpura
Source: PLoS One. 2025 Jun 6;20(6):e0325417. doi: 10.1371/journal.pone.0325417 (PMC12143514; doi:10.1371/journal.pone.0325417)
Supplement: S1 File — (DOCX) [file pone.0325417.s004.docx]

**S1 File. Supplemental Methods.**

WVU Medicine is a hospital system providing care to patients in rural Appalachia within West Virginia, western Maryland, eastern Ohio, and southwestern Pennsylvania. Patients with suspected TTP are transported from affiliate hospitals in the region to a tertiary academic referral medical center in Morgantown, WV which offers hematology care and plasma exchange needed for TTP treatment. ADAMTS13 testing is a send out test. This project was conceptualized with the DMAIC (define, measure, analyze, improve, control) methodology of Six Sigma, and methods are presented as in Improta et al. [1-3] Suspected TTP was at the discretion of the treating physicians.

*Define*. The objective was to improve care for patients with TTP. The aim of the study was to improve TTP therapy, transitions of care, and long-term outcomes. The Critical to Quality characteristic (CTQ) and primary outcome was defined as TTP relapse and TTP-related death (TTP-RRD). For the project TTP relapse was defined as an episode of active TTP with thrombocytopenia requiring therapy occurring in a patient with a history of TTP (either an “exacerbation” or “relapse” as defined by Scully et al).[4, 5] TTP-related death was defined as patient death occurring during the episode of active TTP, or from direct post-TTP sequelae (stroke, myocardial infarction, multi-organ system failure) with no active TTP at the time of death in the medical opinion of the authors. TTP was defined clinically with thrombocytopenia, hemolytic anemia, elevated lactate dehydrogenase (LDH) as previously,[6] with severely reduced ADAMTS13 activity, and absence of alternative etiology. Patients with a prior history of TTP and new acute thrombocytopenia were defined as TTP in the absence of elevated LDH given the possibility of presenting to care earlier due to known TTP history.

Inclusion criteria: age≥18 years, with a diagnosis of new TTP or relapsed TTP (as above). For final retrospective analysis, patients were identified for review from ADAMTS13 testing electronic databases with activity <30% and were assessed by two hematology physicians (SAM and SY) for diagnosis of TTP. Exclusion criteria: age <18, other TMA determined to not be TTP at the discretion of the treating physicians, missing clinical data preventing TTP diagnosis. Patients with TTP in biochemical relapse as outpatients during the intervention, but who had acute TTP outside of the specified study period, were treated on the outpatient TTP Pathway, but were excluded from analyses herein. Patients with congenital TTP or suspected congenital TTP were included. Autoimmune TTP was defined as the presence of ADAMTS13 inhibitor on testing, or normalization of ADAMTS13 activity after immune suppression 1 week or more from last TPE session. TTP-RRD was the primary outcome and would be assessed by log-rank testing, and by proportion analysis at 90 days.

Secondary outcomes were TTP-related death, TTP relapse, use of rituximab in acute TTP, and use of pre-emptive rituximab during clinical remission intended to prevent TTP clinical relapse. Patient distance from the center (>20 miles) was an exploratory contribution to outcome, post-hoc this was changed to 50 miles as only 1 patient was within 20 miles of the center. A 50% reduction in TTP-RRD was defined as intervention success a priori. TTP-RRD was selected as primary outcome because death and TTP relapse are competing events. Analysis of TTP-RRD at the level of episodes was planned given the relapsing nature of TTP. Outcomes would be determined by retrospective chart review. Patients treated on the TTP Pathway were tracked prospectively with TMA team involvement; patients had to be on treatment with disease modifying therapy (TPE or caplacizumab) at hospital discharge and TMA Team had to be informed of the patient by time of discharge to be considered “on pathway.” Full analysis would include patients with TTP as defined above within the study period and would compare those treated on TTP Pathway to usual care. Retrospective analysis would identify potential TTP cases from ADAMTS13 testing results and patients possibly not referred to the TMA Team; patients with activity <30% would have charts reviewed for possible TTP (as above).

The target was inpatient and ambulatory care processes affecting the CTQ. TMA team members were the authors (Hematology, Nephrology, Pharmacy, Transfusion Medicine), and the team leader was a classical hematology physician (SAM). The intervention was planned for a 3 year period (TTP Pathway) to be compared to an equal 3 year retrospective period (usual care). The outcomes of TTP-RRD, TTP relapse, and TTP-related death were defined a priori, as were use of rituximab immune suppression during active TTP, and pre-emptive use of rituximab to obviate TTP clinical relapse.

*Measure.* A process map was done for TTP hospitalization and outpatient care. TMA team members identified the following areas of need: coordination of apheresis line placement, proper selection of catheter type, delineation of subspecialist roles in therapy, collection of ADAMTS13 testing before plasma use, use of immunosuppression including rituximab, timing of rituximab given caplacizumab, duration of TPE, use of novel therapy (caplacizumab), prompt outpatient follow up, clarification of line maintenance roles, patient education on planned therapeutic course, and long-term surveillance to minimize relapse.

Concurrently during October and November of 2019, retrospective chart review was conducted. Patients with TTP were identified from the electronic health record via problem list diagnosis of TTP between 2017 and 2019 and charts were reviewed. TTP relapse in the time period was 50%, and 25% of patients died of TTP. Using the Five Whys method and a check sheet, the following contributors were noted for TTP relapse or death: patient non-adherence to therapy (originating from logistical, substance abuse, or care team communications of plan), short duration of TPE (i.e. stopping soon after platelets reached 150,000/µL), follow up difficulty, lack of ADAMTS13 surveillance, infrequent use of rituximab, and infrequent use of caplacizumab (which was not available for most of the period before the pathway was enacted).[2]

*Analyze.* Causes of TTP-RRD were analyzed with an events and causal factor analysis using an Ishikawa diagram.[2] The authors surmised that the outcome was multifactorial and several changes were needed along the process map to attain the desired change of the CTQ. Using the proactive poka-yoke principle, we identified the following focus areas: having a TTP expert resource (TMA Team[7]), educational materials for care providers, adding caplacizumab to hospital formulary, arrangement of follow up with hematology clinic post-discharge, developing a standardized TPE and rituximab use protocol, and conducting ADAMTS13 active surveillance to detect biochemical relapses before overt TMA has developed[2]. Planned duration of TPE and use of TPE taper was based on author (SAM) prior experience in TTP management at another center with lower apparent TTP relapse rates, as noted with discussion with that center (Dr. Chaturvedi, personal communication, November 15, 2019).

*Improve*. To systematically address outcomes with our limited resources the following changes were adopted: formation of a volunteer TMA team, generation and distribution of resident and consult service education materials (pocket cards), establishing the TPE and immunosuppression protocol with planned duration of TPE and expected rituximab use in acute TTP, arranging outpatient follow up with hematology before discharge from hospital, planned outpatient surveillance of ADAMTS13 activity no less than every 3 months, obtaining caplacizumab on formulary and developing criteria for use.

The TMA team was multi-disciplinary and experienced in TTP management; it was available to the hematology/oncology consult team or medical teams to answer questions but was not a formal consult team. The team members discussed patient care via secure messages in the electronic record, by secure email, or in person. The classical hematologist tracked patients at time of discharge with TTP, hemolytic uremic syndrome (HUS), and atypical HUS (aHUS). Contacting the TMA team was at the discretion of providers with direct patient care. Educational materials in the form of a “pocket guide” were distributed to all hematology/oncology fellows each year of fellowship, and a resident version was available on the institutional resource internal webpage (Supplementary Materials). The fellow guide was discussed with and sent to fellows on 1/9/2020, and was subsequently sent to all fellows each year in July when the new fellowship class begun training. The TTP Pathway was also discussed with fellows during the lecture on thrombocytopenia occurring in the spring of each year. The resident guide was introduced to medicine residents on 2/6/2020 in a lecture on hematologic emergencies, and yearly thereafter. Only the academic center has hematology/oncology fellows in the health system. Any medical staff in the institution could access the institutional resource internal webpage. The TMA Team was engaged by the hematology/oncology consult service or the apheresis service at the academic center, at the discretion of the treating staff providing direct patient care. The effort of those on the TMA Team was not supported by clinical effort, or FTE allotments.

The TPE protocol was as in Rock et al with the following modifications.[8] The TPE protocol used albumin replacement at the beginning of each session and then used fresh-frozen plasma (FFP) in the final half of the procedure to reduce plasma donor exposure.[9] Using the Spectra Optia (Terumo Corporation, Tokyo, Japan), TPE consisted of 1.5 plasma-volume (PV) exchanges daily for 3 days, replacing with 50% albumin and 50% plasma, then 1.0 PV daily replacing with 50%/50% albumin/plasma until platelet count was 150,000/µL (normal range 150,000-400,000/µL) for 3 days consecutively. After this milestone was met, TPE was tapered to every other day, with the goal of no TPE occurring over the following weekend. TPE was expected to use 12-14 sessions, however duration was to be determined at outpatient follow up with the classical hematology clinic. A patient could be ready for hospital discharge when they tolerated a weekend free of TPE without downward trajectory of platelet counts, indicating outpatient Monday-Wednesday-Friday TPE was possible. Prednisone was tapered per physician preference. Rituximab was used at 375mg/m2 for four weekly treatments, and the first rituximab treatment was planned for administration in hospital after the TPE session when no TPE was to be done the following day, to minimize rituximab removal by TPE.[10] All patients were recommended to have rituximab.

Temporary apheresis lines placed emergently for TPE were replaced with tunneled lines prior to hospital discharge. Patients receiving caplacizumab did not require the full number of planned TPE sessions at the discretion of treating physicians, and the decision to use caplacizumab was at the discretion of the treating inpatient physicians; in general caplacizumab was encouraged as per the educational materials (S2 File, S3 File). Caplacizumab was stopped after adequate ADAMTS13 recovery (monitored as above), at the discretion of the treating outpatient hematologist. Tunneled lines were flushed weekly outpatient in the dialysis center, where TPE was conducted. Tunneled lines were removed two weeks after last TPE session, provided platelet count stability.

Laboratory monitoring used twice a week blood counts after TPE cessation for 2 weeks, then once weekly for 4 weeks, then every other week for 4 weeks. Afterwards, blood counts were then checked once a month versus every 3 months at the discretion of the physician. ADAMTS13 activity was checked once a month for 6 months, and then every 2 months for 6 months, and then every 3 months for long term surveillance. If ADAMTS13 activity was observed to drop, frequency of monitoring was changed at the discretion of the physician. Preemptive therapy with rituximab was planned with ADATMS13 activities of 20-30%, or abrupt decreases at the discretion of the treating physician.[11, 12]

*Control*. During the *control* phase of the project TTP-RRD was assessed for patients treated on TTP Pathway by retrospective analysis yearly or when an event occurred. Failures were analyzed with an events and causal factor analysis to determine if intervention process changes were needed consistent with the reactive poka-yoke principle.[2] During the project, ISTH guidelines on TTP were published and encouraged caplacizumab use, thus the TMA team further encouraged its use.[13] A classical hematology pharmacist became available in October 2022 and was a helpful addition to the TMA team, especially with expanded use of caplacizumab and need for medication authorizations and specialty pharmacy coordination. Because of lower-than-expected TTP cases observed during the COVID-19 epidemic that began in March 2020 (compared to prior cases), the prospective project was planned to extend for 1 additional year, 4 years total. At the end of the intervention the full retrospective analysis was conducted as planned a priori.

Final retrospective analysis: Retrospective chart review was conducted to assess outcomes after TTP Pathway implementation. The inclusion criteria and exclusion criteria are listed above under *Define*. The time periods for analysis were January 1, 2016—December 31, 2023. The TTP Pathway became operational on November 11, 2019. Some patients during rollout were not treated on TTP Pathway likely due to unfamiliarity with the Pathway (Supplemental Figure S1). Data were accessed for research purposes on April 10, 2024. Only authors collecting data (SAM and SY) had access to information that could identify individual participants during data collection before de-identification. The project was approved by the WVU Institutional Review Board. This study is presented following STROBE guidelines.[14]

Patients with ADAMTS13 activity test results <30% were identified from the electronic health records and assessed by chart review. Two hematology physicians (SY and SAM) reviewed cases and determined if patients had TTP by consensus, as defined above. Non-TTP etiology of reduced ADAMTS13 activity was also determined by consensus. Clinical information was then abstracted for TTP episodes. Patient episodes treated on the TTP Pathway were identified from the prospectively maintained administrative database.

Non-adherent patients were included in analyses to minimize bias. Patients treated on pathway were recorded prospectively to minimize bias; however not all contacts with the TMA Team were recorded prospectively. To minimize confounding, analysis of TTP-RRD over the project was done for initial TTP presentations. Patients with conditions other than TTP, HUS, and aHUS were not tracked, as focus of this project was TTP treatment and not assessment of the TMA Team itself. Likewise, patients with suspected TTP were not tracked prospectively and “suspected TTP” was not objectively defined for this project. Diagnosis of TTP or not-TTP, and outcomes of relapse or death were determined blinded to Pathway use status. Missing data were omitted for analyses. Reporting TTP associated with COVID-19 infection was added post hoc, and was determined by investigator discretion of temporal relationship between COVID illness and active TTP. Patients were followed from hospital admission with acute TTP until death, relapse, or last known follow up. Patients who were treated with usual care and then entered the TTP Pathway during TTP remission were censored from the usual care group at the time of cross over; they were not included in TTP Pathway analysis as they did not have active TTP at the time of joining the TTP Pathway.

Descriptive statistics were computed with percentages, frequencies, medians, and means as indicated. A priori the primary and secondary outcomes were to be assessed by log-rank testing, and by proportion analysis at 90 days. The null hypothesis (H_0_) was that the TMA Pathway would not affect TTP-RRD at 90 days. Statistical significance was assessed using confidence intervals and effect sizes rather than p-values alone, following contemporary recommendations on statistical reporting. Relative risk (RR) and hazard ratios (HR) with 95% confidence intervals were calculated where applicable. Given the rarity of TTP, a formal power analysis was not feasible, but all available cases meeting inclusion criteria were included. Kaplan-Meier survival analysis and the log rank test were used in comparisons where indicated. Outcomes were analyzed with the test of proportions as indicated or Wilcoxon rank sum test where indicated. All tests were 2-sided and performed at 0.05 level of statistical significance, 95% confidence intervals (95% CI) are reported. Computations utilized STATA 18, (StataCorp. 2023. Stata Statistical Software: Release 18. College Station, TX). The study was planned to analyze approximately equal TTP episodes in the retrospective usual care and prospective TTP Pathway due to comparable time windows, however a formal sample size calculation was not done a priori.

1. Snee, R.D. and R.W. Hoerl, *Leading Six Sigma : a step-by-step guide based on experience with GE and other Six Sigma companies*. Financial Times Prentice Hall books. 2003, Upper Saddle River, NJ: Financial Times Prentice Hall. xxi, 279 p.

2. Westcott, R., *The certified manager of quality/organizational excellence handbook*. Fourth edition. ed. 2014, Milwaukee, Wisconsin: ASQ Quality Press. xxxi, 654 pages.

3. Improta, G., et al., *Lean Six Sigma: a new approach to the management of patients undergoing prosthetic hip replacement surgery.* J Eval Clin Pract, 2015. **21**(4): p. 662-72.

4. Coppo, P., et al., *Predictive features of severe acquired ADAMTS13 deficiency in idiopathic thrombotic microangiopathies: the French TMA reference center experience.* PLoS One, 2010. **5**(4): p. e10208.

5. Scully, M., et al., *Consensus on the standardization of terminology in thrombotic thrombocytopenic purpura and related thrombotic microangiopathies.* J Thromb Haemost, 2017. **15**(2): p. 312-322.

6. Scully, M., et al., *A phase 2 study of the safety and efficacy of rituximab with plasma exchange in acute acquired thrombotic thrombocytopenic purpura.* Blood, 2011. **118**(7): p. 1746-53.

7. Gordon, C.E., et al., *Thrombotic Microangiopathy: A Multidisciplinary Team Approach.* Am J Kidney Dis, 2017. **70**(5): p. 715-721.

8. Rock, G.A., et al., *Comparison of plasma exchange with plasma infusion in the treatment of thrombotic thrombocytopenic purpura. Canadian Apheresis Study Group.* N Engl J Med, 1991. **325**(6): p. 393-7.

9. O'Brien, K.L., et al., *The use of 50% albumin/plasma replacement fluid in therapeutic plasma exchange for thrombotic thrombocytopenic purpura.* J Clin Apher, 2013. **28**(6): p. 416-21.

10. Cheng, C.W., et al., *Therapeutic Plasma Exchange and Its Impact on Drug Levels: An ACLPS Critical Review.* Am J Clin Pathol, 2017. **148**(3): p. 190-198.

11. Westwood, J.P., et al., *Rituximab for thrombotic thrombocytopenic purpura: benefit of early administration during acute episodes and use of prophylaxis to prevent relapse.* J Thromb Haemost, 2013. **11**(3): p. 481-90.

12. Hie, M., et al., *Preemptive rituximab infusions after remission efficiently prevent relapses in acquired thrombotic thrombocytopenic purpura.* Blood, 2014. **124**(2): p. 204-10.

13. Zheng, X.L., et al., *ISTH guidelines for treatment of thrombotic thrombocytopenic purpura.* J Thromb Haemost, 2020. **18**(10): p. 2496-2502.

14. Vandenbroucke, J.P., et al., *Strengthening the Reporting of Observational Studies in Epidemiology (STROBE): explanation and elaboration.* Epidemiology, 2007. **18**(6): p. 805-35.
