# Supplementary material for: The TMA team and TTP pathway improved outcomes in a cohort with Thrombotic thrombocytopenic purpura
Source: PLoS One. 2025 Jun 6;20(6):e0325417. doi: 10.1371/journal.pone.0325417 (PMC12143514; doi:10.1371/journal.pone.0325417)
Supplement: S3 File — Educational guide available to all staff via institutional intranet website, intended for internal medicine resident audience. (PDF) [file pone.0325417.s006.pdf]

TMA: TTP vs aHUS vs DIC

TTP= ↓ PLT, MAHA. Some AKI possible
aHUS= renal failure, mild ↓PLT , MAHA
DIC= ↓PLT, MAHA, in setting of infection or other illness! You can have normal coag tests initially!

Initial History: Ever have TTP before? Need plasma exchange before? Use IV drugs (like Opana)? Use tacrolimus, have organ or BMT?

Initial Labs: ADAMTS13, type and screen, CBC, LDH, CMP, retic, INR, smear, hepatitis B antibody/antigen/core ag. Blood cultures, d-dimer
-Heme fellow can read smear.

Most people with TTP do not have the "pentad" taught in review books!

Compute PLASMIC and DIC scores (https://www.mdcalc.com)

If TTP suspected: Do not administer platelets (↑mortality), even if <10 or for line placement. Place Mahurkar 2 lumen catheter. If in MICU, MICU can place. If on floor, nephrology fellow or Surg/Vasc resident can place. Avoid Arrow catheter, they collapse during PLEX.

Line site: prefer IJ (20cm catheter). If patient unstable femoral is option (24cm catheter).
Line use: confirm placement of IJ. Document line placement.
Follow up labs: Daily CBC, LDH, creatinine.

Daily smear is <u>not</u> helpful

PLEX: plasma exchange should be started as soon as possible, but the line is needed first.
Prednisone: begin 1mg/kg PO daily.
PJP PPX: atovaquone or Bactrim.
Further therapy (rituximab, caplacizumab, eculizumab) will be determined by Hematology.

TTP is lethal (25% mortality 2017-2019)
Untreated TTP is worse (90% mortality)

TTP Expectations: people with TTP require many PLEX sessions. PLEX removes the antibody until the immunosuppression can take effect.
Inform patient that hospitalization will likely be 2 weeks at <u>shortest.</u>
-Patient will need tunneled line before discharge
-Patient will need outpatient PLEX

Usually: PLEX daily until PLT >150 for 3 days in a row. Then we use every-other day PLEX.
-we skip two days over a weekend to see if the disease is stable. Once this occurs, patient <u>could</u> be ready for discharge.
Outpatient transition: Continue M-W-F PLEX.
Follow up in Heme clinic, they will stop PLEX
Weekly line flushes after PLEX done in Cancer Ctr
Roles:
-Hematology: makes diagnosis, evaluates smear
-Nephrology: orders plasma, does PLEX
-MICU/Surg-Vasc: line placement, confirmation
-Blood bank: provides plasma

<u>Call or email the TMA Team if questions!</u>

Samuel Merrill, Ruta Arays, John Gotses, Aaron Shmookler
